# Dural-Based Posterior Fossa Medulloblastoma Mimicking a Petrous Meningioma in Late Adulthood

**DOI:** 10.31486/toj.21.0064

**Published:** 2022

**Authors:** Daniel W. Griepp, Aaron Miller, Jonathan Klein, Ali A. Chaudhri, Stephanie Moawad, Razia Rehmani, Ralph Rahme

**Affiliations:** ^1^ Division of Neurosurgery, SBH Health System, Bronx, NY; ^2^ Department of Radiation Oncology, Albert Einstein College of Medicine and Montefiore Medical Center, Bronx, NY; ^3^ Division of Radiation Oncology, SBH Health System, Bronx, NY; ^4^ Department of Pathology, SBH Health System, Bronx, NY; ^5^ Department of Radiology, SBH Health System, Bronx, NY; ^6^ CUNY School of Medicine, New York, NY

**Keywords:** *Cranial fossa–posterior*, *fibroma–desmoplastic*, *medulloblastoma*, *meningioma*, *neuroectodermal tumors–primitive*

## Abstract

**Background:** Medulloblastoma of the posterior fossa is commonly encountered in pediatric populations but rarely reported in adults. Adult cases of medulloblastoma typically occur in younger patients, tend to arise intra-axially within the cerebellar hemisphere, and usually exhibit classic histopathologic features.

**Case Report:** A 54-year-old male presented with headaches, dizziness, gait instability, and frequent falls that had worsened during the prior 3 months. Imaging and histopathologic analysis revealed extra-axial, dural-based posterior fossa medulloblastoma with desmoplastic/nodular histopathology, mimicking a petrous meningioma. The mass occupied the left cerebellopontine angle. The patient underwent microsurgical gross total resection of the tumor followed by proton beam radiation therapy and was disease-free at 1-year follow-up.

**Conclusion:** Few dural-based posterior fossa medulloblastomas resembling petrous meningiomas have been reported, and to our knowledge, this is the first description of a case to be treated successfully with proton beam therapy in an older adult. Although rare, medulloblastoma can occur extra-axially in the cerebellopontine angle of older adults, potentially mimicking a petrous meningioma. This rare possibility should always be kept in mind, especially if expectant, nonsurgical management is being considered. To optimize outcome, posterior fossa medulloblastoma should be treated with aggressive microsurgical resection followed by radiation therapy. When available, proton beam therapy should be considered.

## INTRODUCTION

Medulloblastoma of the posterior fossa is a common tumor in children but is rarely reported in adults, accounting for less than 1% of primary adult brain tumors.^1-5^ This tumor can be characterized by location, histopathology, and the 2016 World Health Organization immunohistochemistry molecular grouping classification: WNT-activated, SHH-activated, group 3 and group 4.^6^ In adults, posterior fossa medulloblastoma typically occurs before the age of 40 years, often exhibits classic histopathology, and tends to arise intra-axially within the cerebellar hemisphere.^1,7^ Extra-axial medulloblastomas involving the dura of the cerebellopontine angle (CPA) in adults are extremely rare.^3,7^ Such tumors are usually treated with surgery, followed by adjuvant radiation therapy.^2^ We report a case of extra-axial CPA medulloblastoma with desmoplastic/nodular histopathology that mimicked petrous meningioma.

## CASE REPORT

**Figure 1.**
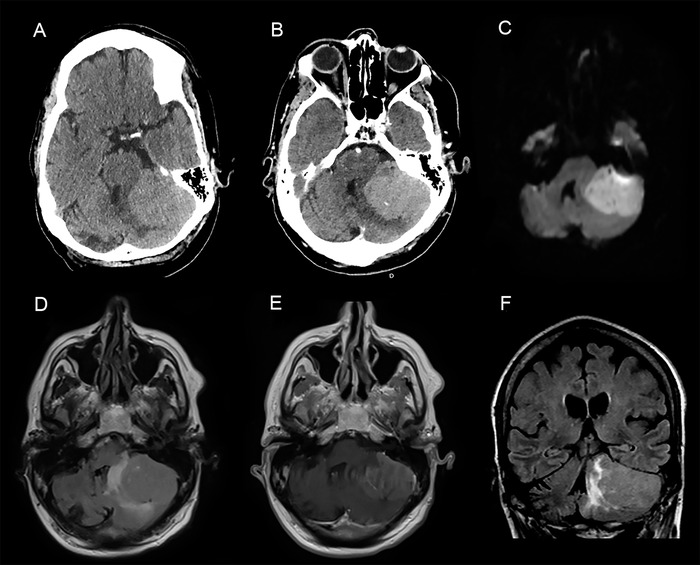
Axial (A) noncontrast and (B) postcontrast head computed tomography images demonstrate a hyperdense, enhancing extra-axial mass lesion of the left cerebellopontine angle. (C, D, E) Axial and (F) coronal brain magnetic resonance images, with and without contrast, demonstrate an enhancing extra-axial posterior fossa mass in the left cerebellopontine angle, exhibiting diffusion restriction (C: diffusion-weighted imaging [DWI]), perilesional edema (F: fluid attenuated inversion recovery [FLAIR]), mass effect, and rightward deviation of the fourth ventricle (F: FLAIR).

A 54-year-old male presented with headaches, dizziness, gait instability, and frequent falls that had worsened during the prior 3 months. The patient had baseline left hemiparesis secondary to an ischemic stroke 4 years prior. Physical examination revealed new left-sided horizontal nystagmus, bilateral dysmetria, and unsteady gait. Head computed tomography and brain magnetic resonance imaging (MRI) revealed a large, extra-axial, dural-based, heterogenous mass with intrinsic cystic foci, occupying the left cerebellopontine angle with surrounding perilesional vasogenic edema and mass effect on the adjacent fourth ventricle (Figure 1). The imaging findings were consistent with an initial working diagnosis of petrous meningioma.

Other differential diagnoses included vestibular schwannomas and the less commonly encountered epidermoid cysts. Given the posterior location of the lesion relative to the vestibulocochlear nerve, the diagnosis of vestibular schwannoma was less likely. In this setting, the diagnosis of petrous meningioma was considered more likely, especially given the appearance of dural attachment to the lesion.

The patient underwent a left lateral suboccipital crani-otomy and microsurgical gross total resection of the lesion via a retrosigmoid approach. The tumor had a well-defined capsule with a broad base of insertion on the petrous dura, including a multitude of dural arterial feeders that were consecutively bipolar coagulated and divided. A few smaller pial arterial feeders, arising from the adjacent cerebellar hemisphere, were also identified and were disconnected in similar fashion. Overall, the tumor appeared to be predominantly extra-axial, with a clear dissection plane separating it from the pial surface of the cerebellum. However, in its most inferomedial portion, the dissection plane was less distinct, as the lesion appeared to infiltrate the adjacent cerebellar parenchyma.

**Figure 2.**
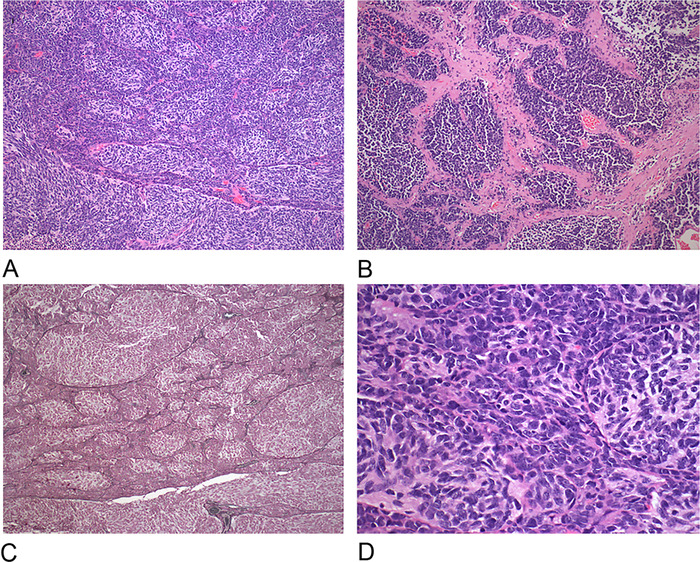
(A) Microphotograph at low-power view shows nodular foci consisting of aggregates of tumor cells with increased cytoplasm and internodular areas containing tumor cells with scant cytoplasm (hematoxylin and eosin [H&E], magnification ×10). (B) Microphotograph at low-power view demonstrates primitive tumor cells embedded in fibrotic/desmoplastic stroma (H&E, magnification ×10). (C) Microphotograph at low-power view reveals increased reticulin deposition within internodular areas (reticulin, magnification ×10). (D) Microphotograph at high-power view shows nodular areas containing tumor cells with increased cytoplasm and internodular areas containing cells with more primitive cytology (H&E, magnification ×40).

Pathologic examination revealed an infiltrating, high-grade neoplasm with sheets of primitive, small, round, and blue tumor cells; prominent nuclear molding; scant cytoplasm; high mitotic activity; and focal nodule formation (Figure 2). Immunohistochemistry showed strong positivity for CD56 and synaptophysin, as well as a markedly increased Ki-67 index, as high as 40% in the internodular areas, consistent with the diagnosis of desmoplastic/nodular medulloblastoma. Molecular analysis using GAB, YAP1, and p53 immunostains showed SHH activation and TP53 wild-type expression.

The patient had a favorable, uneventful postoperative course. His neurologic symptoms resolved, and his gait notably improved. He was discharged to a rehabilitation facility on postoperative day 9.

Given the pathologic findings, a postoperative MRI of the entire craniospinal axis was performed and was negative for metastases. Nonetheless, prophylactic craniospinal radiation therapy was indicated and administered using proton beam therapy for a 3-month treatment course. Treatment consisted of 36 Gy in 18 fractions to the entire brain and spinal canal, followed by additional radiation to the surgical bed in the posterior fossa, totaling 20 Gy in 10 fractions. The total dose to the surgical bed was 56 Gy in 28 fractions.

**Figure 3.**
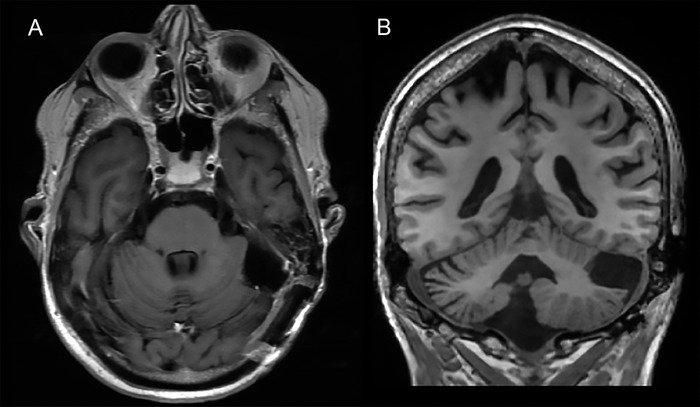
Postcontrast (A) axial and (B) coronal brain magnetic resonance images obtained at 1-year follow-up demonstrate postsurgical changes with no evidence of tumor regrowth or recurrence.

At 1-year follow-up, the patient was completely asymptomatic and, aside from his known baseline left hemiparesis, his neurologic examination was unremarkable. Repeat MRI showed no evidence of tumor regrowth or recurrence (Figure 3). To our knowledge, the use of proton beam therapy in an older adult has not previously been described.

## DISCUSSION

While generally rare in adults, posterior fossa medulloblastomas have been reported primarily in patients in their 20s or 30s rather than those in their sixth decade of life, as in the present case.^1,2,5^ The summary of reported cases in older adults presented in the Table indicates the rarity of medulloblastoma occurring in the CPA in older adults.^3,5,8-13^ Generally, these tumors exhibit classic histopathology and do not involve the dura but rather arise intra-axially within the cerebellar hemisphere.^1,11,12^ In contrast, our patient had an extra-axial, dural-based lesion with desmoplastic/nodular histopathologic features. In the setting of malignancy, desmoplastic reaction is thought to represent a local response to tumor cells, resulting in increased collagen deposition and fibrosis. However, dural-based medulloblastomas tend to differ in that respect. In these tumors, desmoplastic reaction has been shown to involve dense reticulin deposition among tumor cells within the tumor rather than an external response to surrounding cells.^14^ Unlike metastases, melanocytic tumors, or glioblastomas, medulloblastomas are not classic meningioma mimickers.^15^ Favorable features of medulloblastoma (ie, features that suggest lower tumor recurrence risk) include residual tumor <1.5 cm^2^, negative spine MRI and cerebrospinal fluid cytology, classic or desmoplastic histology, and location within the brain that allows for uncomplicated gross total resection.^12,13,16^ High-risk features include bulky residual disease, evidence of leptomeningeal dissemination or distant metastasis, and large cell/anaplastic histology.^12,13^

**Table.** Studies Reporting Cerebellopontine Angle Medulloblastomas Presenting in Adults 30 Years or Older

Study	Age, Sex	Molecular Subgroup	Histology	Tumor Origin	Treatment	Alive at Follow-Up
Becker et al, 1995^5^	32, F	N/R	DNMB	Unknown	N/R	N/R
	52, F	N/R	DNMB	Unknown	N/R	N/R
Mehta and Sharr, 1998^8^	40, M	N/R	DNMB	Brainstem	STR+RT	9 months
Gil-Salú et al, 2004^3^	40, M	N/R	DNMB	Cerebellum	GTR+RT	N/R
Jaiswal et al, 2004^9^	35, M	N/R	Classic MB	N/R	STR	4 months
	40, F	N/R	DNMB	N/R	STR	6 months
	53, M	N/R	Classic MB	N/R	STR	1 month
Fallah et al, 2009^10^	47, M	N/R	Classic MB	Unknown	STR+RT	N/R
Furtado et al, 2009^11^	32, M	N/R	Classic MB	Cerebellum	GTR+RT	N/R
Xia et al, 2019^12^	41, F	WNT	Classic MB	N/R	GTR	9 months
	52, M	SHH	DNMB	N/R	GTR+RT	17 months
Wu et al, 2020^13^	30, M	WNT	Classic MB	Brainstem	GTR+RT+CH	120 months
	34, F	SHH	Classic MB	Cerebellum	STR+RT+CH	91 months
	34, M	SHH	Classic MB	Cerebellum	GTR+RT+CH	110 months
	38, F	SHH	DNMB	Cerebellum	STR+RT+CH	41 months
	42, F	SHH	Classic MB	Cerebellum	GTR+RT+CH	98 months
	45, M	SHH	Classic MB	Cerebellum	STR+RT+CH	Died
Present case, 2022	54, M	SHH	DNMB	Cerebellum	GTR+proton beam RT	12 months

Note: Age is reported in years.

CH, chemotherapy; DNMB, desmoplastic/nodular medulloblastoma; F, female; GTR, gross total resection; M, male; MB, medulloblastoma; N/R, not reported; RT, radiation therapy; SHH, SHH-activated; STR, subtotal resection; WNT, WNT-activated.

Given the location of our patient's tumor in the CPA and its involvement of the lateral hemisphere, gross total resection was likely achieved more readily than if the medulloblastoma had been in the cerebellar vermis. The tumor location within the lateral hemisphere was also less likely to have an attachment to the floor of the fourth ventricle, which would complicate resection given the proximity to the brainstem. Thus, the lateral location of the medulloblastoma may have allowed for a more radical gross total resection and contributed to a more favorable prognosis in this patient. The absence of dissemination to the spine was another favorable factor.

Our review of the literature revealed few adult patients with medulloblastomas mimicking petrous meningiomas.^3,10-12^ The patients were treated with gross total resection followed by conventional radiotherapy. Compared to conventional radiation, which utilizes megavoltage x-rays, proton therapy delivers less radiation to surrounding normal tissues.^17^ This precision allows for curative doses of radiation therapy to the target volumes, while reducing the risk of radiation-related toxicity to normal tissues and radiation-induced malignancy resulting from whole-body radiation exposure.^16,18^ A 2020 study showed that use of proton beam therapy in adults with brain tumors spanning a variety of histopathologies and locations markedly increased between 2004 and 2015.^19^ However, few studies have compared conventional radiation therapy to proton beam therapy in the treatment of medulloblastoma. One study of medulloblastoma treatment using proton beam therapy in young adults specifically demonstrated a lower incidence of radiation-induced toxicity compared with conventional radiation, including weight loss, esophagitis, and low white blood cell count.^20^ However, differences in survival outcomes between patients with medulloblastoma treated with proton therapy and photon therapy have not been comprehensively studied.

In contrast to childhood medulloblastoma for which adjuvant chemotherapy is standard treatment, the role of chemotherapy in the management of medulloblastoma in adults remains controversial. While some studies suggest a survival benefit with postoperative chemotherapy, serious concerns exist regarding the potential toxicity of chemotherapy in adult patients, especially given the lack of randomized controlled trials.^21^ Nonetheless, studies have demonstrated longer survival times for adult patients who were treated with adjuvant chemotherapy for medulloblastoma. However, these studies generally reported outcomes of younger adults with medulloblastoma (average age usually in the late 20s) compared with our patient who was 54 years.^21-23^ Given the rarity of medulloblastoma in older adulthood, the efficacy of adjuvant chemotherapy for this patient group is not well-defined. Treatment of similar tumors with and without adjuvant chemotherapy has been reported with favorable outcomes (Table).^13^ For our patient, the pros and cons of adjuvant chemotherapy were discussed with a neuro-oncologist, and the decision was made not to proceed with this treatment.

## CONCLUSION

Although rare, medulloblastoma can occur extra-axially in the CPA of older adults, potentially mimicking a petrous meningioma. This possibility should be kept in mind, especially if expectant, nonsurgical management is being considered. To optimize outcome, posterior fossa medulloblastoma should be treated with aggressive microsurgical resection followed by radiation therapy. When available, proton beam therapy should be considered. The role of chemotherapy in adult posterior fossa medulloblastoma is not well-defined.

## Notes

*This article meets the Accreditation Council for Graduate Medical Education and the American Board of Medical Specialties Maintenance of Certification competencies for Patient Care and Medical Knowledge.*
